# The COVID-19 pandemic and its impact on tic symptoms in children and young people: a prospective cohort study

**DOI:** 10.1007/s10578-022-01348-1

**Published:** 2022-04-13

**Authors:** Charlotte L Hall, Louise Marston, Kareem Khan, Beverley J Brown, Charlotte Sanderson, Per Andrén, Sophie Bennett, Isobel Heyman, David Mataix-Cols, Eva Serlachius, Chris Hollis, Tara Murphy

**Affiliations:** 1 Institute of Mental Health, School of Medicine, Mental Health & Clinical Neurosciences, NIHR MindTech MedTech Co-operative, University of Nottingham, Innovation Park, Triumph Road, Nottingham, UK; 2 NIHR Nottingham Biomedical Research Centre, Institute of Mental Health, Mental Health & Clinical Neurosciences, University of Nottingham, Innovation Park, Triumph Road, Nottingham, UK; 3 Research Department of Primary Care and Population Health and Priment CTU, University College London, Upper 3rd Floor, Royal Free Campus, Rowland Hill Street, London, UK; 4 UCL Great Ormond Street Institute of Child Health (ICH), 30 Guilford Street, London, UK; 5 Psychological and Mental Health Services, Great Ormond Street Hospital for Children NHS Foundation Trust, Great Ormond Street, London, UK; 6 Centre for Psychiatry Research, Department of Clinical Neuroscience, Stockholm Health Care Services, Karolinska Institutet, Region Stockholm, Stockholm, Sweden

**Keywords:** Covid-19, Tourette syndrome, Tics, Mental health, Children and young people

## Abstract

To understand how children and young people with tic disorders were affected by COVID-19, we compared pre and during pandemic scores on the Yale Global Tic Severity Scale (YGTSS). Participants were young people (*N* = 112; male:78%; 9–17 years) randomised to the control arm of the “ORBIT-Trial” (ISRCTN70758207, ClinicalTrials.gov-NCT03483493). For this analysis, the control arm was split into two groups: one group was followed up to 12-months’ post-randomisation before the pandemic started (pre-COVID group, n = 44); the other group was impacted by the pandemic at the 12-month follow-up (during-COVID group, n = 47). Mixed effects linear regression modelling was conducted to explore differences in YGTSS at 6- and 12-months post-randomisation. There were no significant differences in tic symptom or severity between participants who were assessed before and during COVID-19. This finding was not influenced by age, gender, symptoms of anxiety or autism spectrum disorder. Thus, the COVID-19 pandemic did not significantly impact existing tic symptoms.

## Introduction

The coronavirus (COVID-19) pandemic created a global health crisis resulting in the United Kingdom (UK) government implementing unprecedented societal restrictions from March 2020 to April 2021, including practicing social distancing, lockdown restrictions consisting of home confinement for all but essential activity and wearing of personal protective equipment (PPE) when outside the home. Furthermore, from March-May 2020, school closures were enforced for all but vulnerable children and children of keyworkers, affecting an estimated 80–90% of children [1]. Despite early concerns that these restrictions may exacerbate declining trends in children and young people’s (CYP) mental health in the UK, there is uncertainty as to the actual impact [2, 3], with few published studies examining CYP under 18-years [4] or CYP with existing mental health conditions.

The impact of COVID-19 is difficult to calculate being confounded by several key factors, including the evidence of existing decline in CYPs mental health pre-pandemic [2], restricted provision of some mental health services during the pandemic [4] and the lack of pre/post-pandemic measures in order to assess change. Thus, it is perhaps unsurprising that the existing evidence is heterogeneous [5].

Early findings indicated that lockdown was associated with poor mental health [6, 7]. Probable mental health conditions in the UK rose from 10.8% to 2017 to 16% in July 2020 [8], with respondents from lower socioeconomic groups reporting the greatest mental health deterioration [9]. One of the few studies with pre/post pandemic data (conducted outside the UK) indicated a moderate increase in depressive symptoms during lockdown [10]. However, UK based surveys showed that young people reported feeling happier during summer 2020 [11], with no change in anxiety, depression or well-being. Indeed, those who were struggling pre-lockdown made significant improvements in these three areas [5, 12].

Although during early lockdown, it was intuitively believed that young people with an existing mental health condition would be particularly vulnerable to the negative mental health impact of COVID-19 [13] and school closures [14], there was also evidence that some of this group benefited from being released from the complexities and stressors associated with school attendance [15]. However, to date, there have been limited studies into the impact of lockdown on children with specific mental health conditions and neurodevelopmental disorders. Amongst other conditions, the UK National Health Service (NHS) have identified young people with mental health conditions, including neurodevelopmental disorders (such as attention deficit hyperactivity disorder [ADHD] and autism spectrum disorder [ASD]) as being vulnerable to the negative impact of COVID-19 [13]. No evidence is available on these conditions, but research suggests young people with anxiety are more likely to have been significantly impacted [16]. Given the associated changes in routine, young people with ASD may also have been disproportionately affected, however, the available evidence is varied with some reporting improvement in symptoms and others deterioration [17].

Young people with tic disorders may be particularly affected by the pandemic. Tics are sudden, repeated, non-rhythmic motor or vocal movements. Tourette syndrome, a form of tic disorder, is often diagnosed if two or more motor tics (e.g., blinking and shoulder shrugging) and at least one vocal tic (e.g., clearing the throat) have been present for at least one year. The condition is highly comorbid with other neuropsychiatric disorders; 85% of young people with a tic disorder will also have obsessive-compulsive disorder, ADHD, depression, anxiety and/or ASD [18]. Statistics pre-pandemic suggest that Tourette syndrome and chronic tic disorders affect approximately 1% of young people and are associated with significant distress, impairment and reduced quality of life [19]. There are specific consequences of COVID-19 that are likely to impact on patients with tics, including pandemic-related anxiety, confinement, restrictions on physical exercise [20], or the development or increase of “COVID-type” tics (such as coughing) [21]. Anecdotally, clinicians in the UK have reported a significant increase in new tic and “tic-like” cases, as well as an increase in tics in young people diagnosed prior to the pandemic, particularly in teenage girls [22–24]. These tics appear to be atypical in nature, with an acute onset and a circumscribed range of complex vocal and motor tics, often in the absence of what are typically more common simple tics. These previously unusual presentations were uncommon prior to the pandemic and are referred to in the literature as functional tics (a type of functional neurological disorder) due to the fact that they are caused by an abnormality of the functioning of the nervous system rather than an underlying disease and may often be related to psychological or social stress [25, 26].

Beyond ad-hoc clinical reports, there has been limited research into the impact of COVID-19 on tics. A COVID survey of 178 adults with a tic disorder in Europe and North America found that approximately half (48%) of respondents reported a perceived worsening in their tic symptoms, 8% reported tic improvement, with the remainder (44%) reporting no change in tic symptoms. Adults reporting a worsening of symptoms were younger and with more severe existing tics, although no impact of comorbidity was found [27]. To the best of the authors’ knowledge, to date, the only study in young people with tics was a survey conducted in Italy with parent respondents. The survey found that 67% of parents reported worsening of their child’s clinical symptoms since COVID-19, with 20.5% reporting an improvement and 6.7% reporting no change. The findings were not influenced by parental health or their economic situation [23]. However, these studies are limited in that they rely on perceived changes in symptoms since the pandemic, with no direct pre/post pandemic standardised assessment, or in the clinical reports, they are based on individual case studies. To support healthcare professionals in providing appropriate care, there is a need to better understand the impact of COVID-19 and its associated lifestyle changes on CYP with tics in a population-based study. It is also important to identify CYP who might be most vulnerable to developing changes in tics in response to significant environmental stressors [16].

Data from the Online Remote Behavioural Intervention for Tics (ORBIT) randomised controlled trial [28] is uniquely placed to address this question. The trial recruited CYP in England, who were randomised to receive either a remotely delivered behavioural intervention for tics (the intervention group) or remotely delivered psychoeducation (the control group). Participant enrolment and the first baseline measures began in May 2018 and ended September 2019. Participants were followed-up at 3, 6 (phase 1), 12 and 18-months (phase 2) post randomisation. Measures of tic symptoms and severity were recorded using the Yale Global Tic Severity Scale [29] (YGTSS) by trained assessors at each time point. The findings from phase 1 are published and demonstrate that the behavioural intervention was clinically effective and superior to the control arm in improving tic symptoms [30]. For approximately half the participants, the 12-month follow-up assessment coincided with the COVID-19 time period (henceforth, *during COVID-19* group), for the other half, the 12-month follow-up was in pre-pandemic times (henceforth, *pre-COVID-19* group). Utilising YGTSS data from the control group only (to minimise the confounding impact of treatment on symptom course), we aimed to understand changes in tic symptoms in CYP with a pre-existing tic disorder during the COVID-19 pandemic compared to a group of CYP who had been followed-up pre-pandemic. Although the study was not originally designed to assess this, in this post-hoc analysis we investigated whether tic severity and impairment in CYP with a tic disorder was different between the pre-COVID-19 and during COVID-19 groups. We also investigated for any differences in tic complexity. Tic complexity was specifically assessed as a possible indicator of functional tics. Although similar to classical tics, functional tics tend to be more complex in presentation and do not typically follow the rostro-caudal gradient [31]. We hypothesised that, if COVID-19 had impacted on tics, we would see a difference in YGTSS scores between the pre-COVID-19 and during COVID-19 groups. We also further sought to explore whether this finding was influenced by age, sex, or comorbid symptoms of anxiety and ASD, given suggestion in the literature to date that neurodevelopmentally complex children may have been most vulnerable to increased/changes in tics during the COVID-19 pandemic.

## Method

### Participants

The sample were *N* = 112 participants enrolled into the ORBIT Trial [28] and randomised to the control (psychoeducation) group. The ORBIT Trial was a two-arm, parallel group, single-blind, multi-centre study conducted in two study sites in England. The aim of the trial was to investigate the clinical and cost-effectiveness of remotely-delivered, therapist-supported behavioural therapy for tics. The trial was prospectively registered with ISRCTN (ISRCTN70758207) and ClinicalTrials.gov (NCT03483493). Participants in the psychoeducation group received a 10-week, remotely delivered, therapist-supported intervention which focussed on tic education. Content covered included the definition, history, prevalence, and aetiology of tics. No information on tic control was provided. In contrast, the intervention arm received information on how to control tics using exposure and response prevention techniques. Given the potential confounding this may represent when judging the course of tics over time, only participants in the psychoeducation group (control) were used for this analysis. Participants were followed at 3-, 6-, 12- and 18-months post-randomisation.

Eligible participants were children aged 9–17 years with a moderate/severe tic disorder, defined as scores on the YGTSS [29] Total Tic Severity Score (TTSS) of > 15, or > 10 if only motor or vocal tics were present in the last 7 days. Exclusion criterion were: engaged in structured behavioural intervention for tics within the preceding 12 months or about to start; changed (i.e. starting or stopping) medication for tics within the previous two months; alcohol/substance dependence, psychosis, suicidality or anorexia nervosa, suspected moderate/severe intellectual disability, immediate risk to self or others; parent/carer of child unable to speak or read/write English.

Participants were recruited at (i) one of 16 patient identification child and adolescent mental health services (CAMHS) or community paediatric clinics in England, (ii) the two study sites, or (iii) by self-referral via Tourettes Action (UK tic disorder charity) website or the study website. Informed consent was obtained from all participants. Where the child was under 16 years old, parents provided written consent for their child’s participation, verbal or written assent was also gained from the young person. Where the child was 16 years old or over, both the parent and young person provided written consent. Ethical and Health Research Authority (HRA) approval was granted by North West Greater Manchester Research Ethics Committee on 23 March 2018 (ref.:18/NW/0079). Details of the trial protocol and its primary outcome have been published [28, 30].

## Measures

### Yale Global Tic Severity Scale (YGTSS)

The YGTSS is an investigator-based semi-structured interview completed with the parent and the young person. The scale focusses on motor and vocal tic number, frequency, intensity, complexity, and interference to give an overall severity rating, and tic related impairment over the previous week. The YGTSS symptom checklist comprises 46 tics, including 12 simple motor tics (e.g., eye blinking), 19 complex motor tics (e.g., facial expressions), seven simple vocal tics (e.g., coughing), and eight complex vocal tics (e.g., words), with four of these items designated on the instrument as “other” symptoms. Five index scores can also be obtained: Total Motor Tic Score (sum of all motor tics, 0–25), Total Phonic Tic Score (sum of all phonic tics, 0–25), YGTSS-TTSS (sum of motor and phonic tics, 0–50), Overall Impairment Rating (0–50), and Global Severity Score (0-100). The Overall Impairment Rating is rated on a 50-point scale anchored by 0 (*no impairment*) and 50 (*severe impairment*). The complexity item is scored as 0 to 5 points for motor and vocal tics separately, so a total of 0 to 10 points for both combined can be obtained. For all indices, higher scores mean greater presence/impact of tics. For this study, we used the YGTSS overall impairment score, the complexity score, and the YGTSS-TTSS.

All assessors were trained in conducting YGTSS by a clinical expert (author TM). Agreement with the expert rater was established at the start of the trial and monitored every 6-months throughout the trial. The YGTSS was conducted face-to-face at baseline and via videoconferencing or telephone at each follow-up stage. The assessor was blind to the arm allocation within the trial.

### Social Communication Questionnaire (SCQ)

The social communication questionnaire (SCQ) [32] was used to further understand autistic behaviors from the age of 4-years to current functioning. The SCQ was completed by the parents at baseline. It consists of 40 items and total scores range from 0 to 39, with higher scores indicating more autistic behaviour. The scale has been well validated to show good sensitivity and specificity for ASD [33], and a cut-off of > 15 is considered to reliably reflect likely autism [32].

### Spence Child Anxiety Scale (SCAS)

The Spence Child Anxiety Scale (SCAS) [34] was completed by the young person at baseline to assess anxiety. The scale consists of 44 items; 38 items reflect specific symptoms of anxiety disorders, including panic attack and agoraphobia (9 items), separation anxiety disorder (6 items), obsessive–compulsive disorder (6 items), social phobia (6 items), generalized anxiety disorder (6 items), and physical injury fears (5 items). The remaining six items are positive filler questions to reduce responder bias (these are not scored). Each item was rated on a 4-point scale ranging from 0 (*never*) to 3 (*always*).

### Index of Multiple Deprivation (IMD)

Index of Multiple Deprivation (IMD) is a relative measure of deprivation across seven different domains: income deprivation; employment deprivation; education, skills and training deprivation; health deprivation and disability; crime; barriers to housing and services and living environment deprivation [35]. Participants’ six-digit postcode, which was captured at the baseline assessment, was input into the UK government’s website [35], where a rank of deprivation associated with participants’ area of residence was calculated from 32,844 small areas or neighbourhoods in England, with higher ranks indicating greater deprivation. Ranks were re-coded into quintiles in this study with 1 being most deprived and 5 being least deprived.

### Data analysis

In order to investigate the impact of COVID-19, we noted whether follow-up data were obtained prior to COVID-19 or during COVID-19. Only data up to 12 months’ post recruitment were included in the analysis, as by 18-months, the number unaffected by the COVID period was too small for meaningful analysis.

Baseline statistics (demographics and the outcomes) were calculated overall and by COVID-19 timing at 12 months.

Categorical variables were summarised as frequencies and percentages, and chi square tests were used to determine whether there was a difference between characteristics and timing of 12-month data collection. For continuous variables mean (SD) or median (IQR) were used depending on distribution and accompanied by one-way ANOVA, two sample t-test or Kruskal-Wallis test as appropriate.

For the statistical modelling, first, mixed effects linear regression modelling was carried out for the three outcomes: YGTSS-TTSS, impairment score and complexity score using data at 6 and 12 months in the outcome (controlling for baseline outcome, age, sex, SCAS and SCQ). The SCQ was dichotomised to ≥ 15 versus < 15. The models included a time variable and a time varying variable to indicate whether the data were collected pre or during COVID-19. Data from 3 months were not included as there was no variation in COVID-19 timing between the two groups at this follow-up (all data were collected before COVID-19). The objective of this longitudinal analysis was to explore any changes in tic symptoms over time and between the pre and during COVID groups.

Four models were calculated: (1) including time, COVID timing and baseline of the outcome, (2) fully adjusted with the variables in model 1 plus age, sex, SCAS, SCQ. Models 3 and 4 were the same as 1 and 2, except they also included interactions between time and COVID timing.

Following this, cross-sectional analyses at 12 months only were calculated using multiple linear regression. The objective of this analysis was to further test the findings of the longitudinal analysis and gain further insight into how the pre and during COVID groups may have differed at the 12-month time point. For this, two models were conducted: (1) including COVID timing and baseline score of the outcome, (2) fully adjusted including the same variables as in model 1, plus age, sex, SCAS score and dichotomised SCQ. The statistical analysis plan for the study was made publicly available ahead of conducting the analysis. (see Pandemic_and_tics_ORBIT_study_proposal_2021_FINAL.pdf (institutemh.org.uk)).

Additionally, we conducted a further unplanned sensitivity analysis, excluding 12-month assessments in March 2020 to investigate whether the patterns remained consistent removing the time-period when COVID-19 lockdown was first instigated in the UK. All analyses were carried out using Stata version 16.

## Results

In total, 112 participants were consented and randomised to the control arm of the ORBIT Trial [30]. Table 1 shows the total sample characteristics and the mean age of the sample of 12.4 years (SD = 2.1);78% of participants were male and 88% white. The mean YGTSS-TTSS fell within the moderate to severe range: 28 (SD = 7) at baseline, 25 (SD = 8) at 6 months and 25 (SD = 7) at 12 months. All data at baseline were collected before the COVID-19 pandemic, and only 5% of the data at 6 months were collected during the pandemic (for the during COVID-19 group).

**Table 1:** Total sample (N = 112) characteristics

Characteristic	Mean or n/N	(SD) or %
**Baseline**		
Age	12.4	(2.1)
Male	87/112	78
White	99/112	88
Index of multiple deprivation quintiles (where 1 is most deprived)		
1 (most deprived)	12/105	11
2	16/105	15
3	26/105	25
4	18/105	17
5 (least deprived)	33/105	31
YGTSS-TTSS	28	(7)
YGTSS Impairment score	23	(10)
YGTSS complexity score	4.5	(2.4)
Social Communication Questionnaire score median (IQR)	7	(3, 12)
Social Communication Questionnaire ≥ 15	19/112	17
SCAS median (IQR)	28	(18, 40)
**6 months**		
YGTSS data collected after COVID-19 lockdown	5/93	5
YGTSS-TTSS	25	(8)
YGTSS Impairment score	17	(11)
YGTSS complexity score	4.2	(2.4)
**12 months**		
YGTSS data collected after COVID-19 lockdown	47/91	52
YGTSS-TTSS	25	(7)
YGTSS Impairment score	17	(11)
YGTSS complexity score	3.8	(2.4)

*Note.* SCAS = Spence Child Anxiety Scale. SCQ = Social Communication Questionnaire. YGTSS = Yale Global Tic Severity Scale. TTSS = Total Tic Severity Score

Of the total *N* = 112, 44 (39%) were in the pre-COVID-19 group (their 12-month follow-up was not during the COVID-19 time period), and 47 (41%) were in the during COVID-19 group (their 12-month follow-up was during COVID-19). Data from the remaining 21 (18%) participants were missing at the 12-month follow-up. The timing for the data collection of the 12-month data in the pre-COVID group was 17th May 2019 to 19th March 2020. The timing for the 12 month during-COVID group data was 23rd March 2020 to 29th October 2020.

Table 2 shows that baseline characteristics, including severity and impairment of tics, were similar between those whose 12-month data were collected pre-COVID-19, during COVID-19 or have 12-month data missing.

**Table 2:** Sample characteristics by 12-month assessment timing in relation to COVID-19 and presence of 12-month tic data

Characteristic	Pre-COVID group (n = 44)		During COVID group (n = 47)		Missing 12 month data group (n = 21)		
	**Mean or n/N**	**(SD) or %**	**Mean or n/N**	**(SD) or %**	**Mean or n/N**	**(SD) or %**	**p-value**
**Baseline**							
Age	12.3	(2.0)	12.5	(2.3)	12.4	(2.1)	0.934
Male	35/44	80	33/47	70	19/21	90	0.167
White	39/44	89	42/47	89	18/21	86	0.908
Index of multiple deprivation quintiles (where 1 is most deprived)							0.555
1 (most deprived)	5/39	13	6/45	13	1/21	5	
2	4/39	10	6/45	13	6/21	29	
3	11/39	28	10/45	22	5/21	24	
4	5/39	13	8/45	18	5/21	24	
5 (least deprived)	14/39	36	15/45	33	4/21	19	
YGTSS-TTSS	28	(7)	30	(7)	27	(9)	0.338
YGTSS Impairment score	21	(10)	25	(10)	21	(9)	0.097
YGTSS complexity score	4.5	(2.3)	4.9	(2.4)	3.9	(2.7)	0.309
Social Communication Questionnaire score median (IQR)	7	(3, 11)	7	(3, 13)	5	(4, 9)	0.914
Social Communication Questionnaire ≥ 15	6/44	14	10/47	21	3/21	14	0.585
SCAS median (IQR)	26	(15, 36)	34	(20, 46)	24	(16, 34)	0.110
**12 months**							
YGTSS-TTSS	24	(7)	26	(7)			0.202
YGTSS Impairment score	18	(11)	17	(11)			0.691
YGTSS complexity score	3.6	(2.3)	4.0	(2.5)			0.423

*Note.* SCAS = Spence Child Anxiety Scale. SCQ = Social Communication Questionnaire. YGTSS = Yale Global Tic Severity Scale. TTSS = Total Tic Severity Score

The results of the mixed model analyses are presented in Table 3 and show that there was no significant effect of time, group (pre or during COVID) or interaction effect, for any of the outcomes from the four models used. Thus, COVID-19 did not significantly impact on tic severity, impairment, or complexity. This finding was not influenced by age, gender, or baseline presence of anxiety (SCAS scores) or ASD status (SCQ scores).

**Table 3:** Longitudinal analysis exploring the impact of COVID-19 on tics

	Model 1		Model 2		Model 3		Model 4	
	**Coefficient**	**95%CI**	**Coefficient**	**95%CI**	**Coefficient**	**95%CI**	**Coefficient**	**95%CI**
**YGTSS-TTSS**								
Main effect of time (12 months)	0.23	(-1.25, 1.72)	0.26	(-1.23, 1.74)	-0.10	(-1.69, 1.49)	-0.05	(-1.65, 1.54)
Main group effect (Assessment during COVID-19)	-0.34	(-2.28, 1.60)	-0.41	(-2.36, 1.53)	-2.66	(-7.14, 1.82)	-2.55	(-7.06, 1.96)
Interaction effect (12 months and during COVID-19)					2.72	(-2.00, 7.44)	2.51	(-2.26, 7.27)
P-value for interaction					0.259		0.303	
YGTSS-TTSS at baseline	0.71	(0.57, 0.85)	0.70	(0.55, 0.84)	0.71	(0.58, 0.85)	0.70	(0.55, 0.84)
Age			-0.04	(-0.52, 0.43)			-0.02	(-0.50, 0.46)
Female			0.32	(-1.99, 2.64)			0.22	(-2.09, 2.53)
SCAS at baseline			0.02	(-0.03, 0.08)			0.02	(-0.04, 0.08)
SCQ ≥ 15			0.55	(-2.01, 3.12)			0.46	(-2.09, 3.01)
**YGTSS tic impairment**								
Main effect of time (12 months)	1.99	(-0.92, 4.90)	1.97	(-0.94, 4.87)	2.43	(-0.66, 5.53)	2.34	(-0.76, 5.44)
Main group effect (Assessment during COVID-19)	-3.06	(-6.77, 0.65)	-2.96	(-6.64, 0.73)	-0.13	(-8.70, 8.44)	-0.53	(-9.14, 8.07)
Interaction effect (12 months and during COVID-19)					-3.47	(-12.55, 5.61)	-2.89	(-12.06, 6.28)
P-value for interaction					0.454		0.536	
Impairment score at baseline	0.33	(0.16, 0.50)	0.33	(0.13, 0.52)	0.33	(0.16, 0.50)	0.33	(0.13, 0.52)
Age			0.26	(-0.57, 1.10)			0.24	(-0.61, 1.08)
Female			1.95	(-2.11, 6.02)			2.07	(-2.03, 6.17)
SCAS at baseline			-0.01	(-0.13, 0.10)			-0.01	(-0.12, 0.10)
SCQ ≥ 15			-3.87	(-8.39, 0.64)			-3.76	(-8.32, 0.79)
**YGTSS tic complexity**								
Main effect of time (12 months)	-0.29	(-0.80, 0.22)	-0.29	(-0.81, 0.22)	-0.38	(-0.93, 0.17)	-0.36	(-0.91, 0.19)
Main group effect (Assessment during COVID-19)	-0.15	(-0.80, 0.49)	-0.16	(-0.81, 0.49)	-0.77	(-2.28, 0.74)	-0.61	(-2.13, 0.90)
Interaction effect (12 months and during COVID-19)					0.73	(-0.88, 2.33)	0.54	(-1.08, 2.16)
P-value for interaction					0.375		0.515	
YGTSS tic complexity score at baseline	0.68	(0.55, 0.80)	0.68	(0.55, 0.81)	0.68	(0.55, 0.80)	0.68	(0.55, 0.81)
Age			-0.08	(-0.22, 0.07)			-0.07	(-0.22, 0.07)
Female			0.17	(-0.54, 0.87)			0.14	(-0.57, 0.85)
SCAS at baseline			0.00	(-0.01, 0.02)			0.00	(-0.01, 0.02)
SCQ ≥ 15			0.46	(-0.32, 1.25)			0.45	(-0.34, 1.23)

*Note.* SCAS = Spence Child Anxiety Scale. SCQ = Social Communication Questionnaire. YGTSS = Yale Global Tic Severity Scale. TTSS = Total Tic Severity Score

The findings from the cross-sectional analysis at 12 months are presented in Table 4 and confirm the findings of the longitudinal analysis; there was no statistically significant difference in tics between those who were assessed before and during COVID-19 and this was not influenced by age or gender, or baseline symptoms of anxiety or ASD.

**Table 4:** Cross-sectional analysis at 12 months only

	Model 1		Model 2	
	**Coefficient**	**95%CI**	**Coefficient**	**95%CI**
**YGTSS-TTSS**				
Between group effect (during COVID-19)	0.87	(-1.55, 3.28)	0.66	(-1.86, 3.18)
Total tic score at baseline	0.67	(0.49, 0.85)	0.65	(0.45, 0.84)
Age			-0.00	(-0.62, 0.61)
Female			0.94	(-2.02, 3.90)
SCAS at baseline			0.01	(-0.07, 0.09)
SCQ ≥ 15			1.03	(-2.95, 13.41)
**YGTSS Tic impairment**				
Between group effect (during COVID-19)	-1.87	(-6.54, 2.80)	-1.79	(-6.42, 2.85)
Impairment score at baseline	0.24	(0.00, 0.47)	0.28	(0.01, 0.55)
Age			-0.20	(-1.33, 0.92)
Female			5.98	(0.54, 11.42)
SCAS at baseline			-0.05	(-0.20, 0.11)
SCQ ≥ 15			-5.75	(-11.71, 0.22)
**YGTSS Tic complexity**				
Between group effect (during COVID-19)	0.15	(-0.66, 0.97)	0.06	(-0.79, 0.91)
Tic complexity score at baseline	0.61	(0.44, 0.79)	0.61	(0.43, 0.79)
Age			-0.06	(-0.27, 0.15)
Female			0.04	(-0.96, 1.04)
SCAS at baseline			0.01	(-0.02, 0.03)
SCQ ≥ 15			0.63	(-0.46, 1.73)

*Note.* SCAS = Spence Child Anxiety Scale. SCQ = Social Communication Questionnaire. YGTSS = Yale Global Tic Severity Scale. TTSS = Total Tic Severity Score

A further, unplanned, sensitivity analysis excluding 12-month data collected in March 2020 (removing *n =* 8/44 in the pre-COVID group and *n =* 4/46 in the during-COVID group) revealed no statistically significant changes in the findings for either the longitudinal analysis (Table 5) or the cross-sectional analysis (Table 6).

**Table 5:** Longitudinal analysis of YGTSS-TTSS exploring the impact of COVID-19 on tics without 12 month assessments in March 2020

	Model 1		Model 2		Model 3		Model 4	
	**Coefficient**	**95%CI**	**Coefficient**	**95%CI**	**Coefficient**	**95%CI**	**Coefficient**	**95%CI**
Main effect of time (12 months)	0.55	(-0.94, 2.04)	0.56	(-0.93, 2.05)	0.14	(-1.48, 1.76)	0.18	(-1.44, 1.80)
Main group effect (Assessment during COVID-19)	0.31	(-1.64, 2.26)	0.26	(-1.70, 2.21)	-2.07	(-6.29, 2.16)	-1.97	(-6.23, 2.28)
Interaction effect (12 months and during COVID-19)					2.85	(-1.62, 7.33)	2.68	(-1.84, 7.20)
P-value for interaction					0.211		0.245	
YGTSS-TTSS at baseline	0.72	(0.59, 0.86)	0.71	(0.56, 0.86)	0.73	(0.59, 0.86)	0.71	(0.57, 0.86)
Age			-0.06	(-0.54, 0.41)			-0.03	(-0.51, 0.44)
Female			0.36	(-1.95, 2.66)			0.26	(-2.03, 2.54)
SCAS at baseline			0.02	(-0.03, 0.08)			0.02	(-0.04, 0.08)
SCQ ≥ 15			0.33	(-2.22, 2.87)			0.23	(-2.29, 2.75)

*Note.* SCAS = Spence Child Anxiety Scale. SCQ = Social Communication Questionnaire. YGTSS = Yale Global Tic Severity Scale. TTSS = Total Tic Severity Score

**Table 6:** Cross-sectional analysis of the YGTSS-TTSS without those assessed in March 2020 at 12 months only

	Model 1		Model 2	
	**Coefficient**	**95%CI**	**Coefficient**	**95%CI**
Between group effect (during COVID-19)	1.88	(-0.53, 4.30)	1.66	(-0.87, 4.18)
Total tic score at baseline	0.69	(0.51, 0.87)	0.67	(0.47, 0.87)
Age			-0.06	(-0.67, 0.56)
Female			1.28	(-1.65, 4.21)
SCAS at baseline			0.02	(-0.06, 0.09)
SCQ ≥ 15			0.54	(-2.64, 3.72)

*Note.* SCAS = Spence Child Anxiety Scale. SCQ = Social Communication Questionnaire. YGTSS = Yale Global Tic Severity Scale. TTSS = Total Tic Severity Score

## Discussion

Given the uncertainty around the impact of COVID-19 on specific mental health conditions for CYP, combined with the lack of pre/during-pandemic data to reliably investigate the impact, here we sought to utilise data from the ORBIT-Trial to directly assess change on tics throughout COVID-19. The findings did not reveal any significant differences in tic severity, impairment or complexity symptoms or impact for children and young people followed over a 12-month period pre and during the COVID-19 pandemic. This finding was not affected by age or gender, or baseline symptoms of anxiety or ASD. Furthermore, the findings were not significantly impacted by removing 12-month data collected in March 2020 (when COVID-19 lockdown was first initiated in the UK). To our knowledge, this is the first evidence using pre-pandemic baseline and follow-up scores to investigate the impact of COVID-19 on CYP with a tic disorder. However, it is important to note that these analyses were post-hoc and the study was not specifically designed to address the question.

The data neither indicated an improvement nor worsening of tics in either of the two groups. It is possible that the school closures and the attenuation of associated school anxiety for young people with tics may have acted as mitigating action offsetting any lockdown or disease-related anxiety [15]. However, given that school closures were at times localised (i.e. due to bubble contamination), it is not possible to accurately know which follow-up data were collected during extreme measures (i.e. school closures, localised lockdowns). Studies are emerging demonstrating improvements, deterioration and no change in child mental health related to the pandemic – with it being likely that some vulnerable groups may be differentially affected [5, 12]. However, although there is a recognised lack of evidence from condition-specific studies, the limited available data on tics have shown a perceived increase in existing symptoms [23, 27]. Our findings, which rely on blind rater assessments of tics do not support this. It is possible this discrepancy in findings reflects the age of participants, with one of the two existing studies being conducted in adults [27], and the other reported parents’ perceptions of their child’s symptoms [23]. It is possible that young people with tics fared better than adults during the pandemic, which may in part result from reduced academic stress and increased family time due to school closures [36]. It is also possible that the differences reflect cultural and cross-country differences in the approach to the pandemic. The previous data were collected in Europe and North America, whereas our data were solely collected in England. Each country took a different approach to the timing, rules imposed and media strategy during COVID-19 and lockdown which may have differentially impacted on tic symptoms. Finally, these studies collected opinions on tic symptoms, rather than actual observed and quantifiable data from an independent rater.

It is interesting to note that our findings are not consistent with clinician reports of a perceived increase in the number of tics, both in children and young people with existing tics, as well as new cases [22]. It is possible that this perception is driven predominately by an increase in the number of new tic cases including functional tics, rather than exacerbation of tics in existing cases. Furthermore, given the delay in seeking help during the pandemic, it is difficult to assess whether any increase in referrals represents an increase in cases or simply reflects congestion caused by COVID-19 delays [5]. Our study is limited in only being able to assess impact in children with existing tics, and thus we could not address this. It is also important to note that increases have been predominately noted in teenage girls [22, 24], whereas our sample was predominately male with an average age of 12-years. The number of females was likely too small to conduct a meaningful analysis, however, our modelling did not reveal any differences between males and females between the pre and during COVID group. Furthermore, as a large proportion of our sample were from the least deprived areas of England, thus more advantaged families may have been over-represented in our sample. Given the noted inequality of COVID-19 impact across different socio-economic groups [5], our findings may not be generalisable to more deprived populations. Unfortunately, due to the small numbers in the most deprived groups it was not possible to explore this statistically in this sample.

Our study was not specifically designed to address the research question but offers some considerable strengths over previous studies. Tic symptoms were rated by trained, independent professionals. These data also provide a unique opportunity to follow a cohort of children, recruited prior to COVID-19, approximately half of whom had and had not been affected by the COVID-19 pandemic at a 12-month follow-up. The participants were recruited nationally across England and are broadly representative of the typical population usually referred for tics, in that they were pre-teens, predominately male and had moderate-severe tics. Thus, it is likely that our sample is reflective of a typical population seen in child mental health services with higher proportion of males, and high levels of co-occurring neurodevelopmental conditions. One limitation of the study is that, by definition, the two groups did not participate in the study at the same time (the recruitment for ORBIT-Trial was conducted over 18 months) meaning that other factors, not measured in the study, could have influenced the results.

The participants in our study did not receive any behavioural therapy for tics or start medication for tics during the first six months of the study. However, they were involved in a tic-focused control intervention which may have offered them some benefit beyond tic reduction. Participants were also able to start therapy or tic medication outside of the trial after six months; however, given the difficulty accessing services during COVID-19 [3], combined with the difficulties in accessing tic treatment even pre-COVID [37], it is unlikely that actual COVID-19 related increases in tics were masked by appropriate support being accessed elsewhere. Indeed, the converse is possible, that beyond the trial, due to other clinical groups requiring additional support, that young people with tics may have been disadvantaged in an already provision-limited system [37].

The findings from this post-hoc analysis of the ORBIT trial suggest that COVID-19 may have had little impact on clinician-rated tic symptoms and impairment in children and young people with pre-existing tics in England. Whilst there may be patients with tic disorders who did experience an increase in symptoms, for which they will require appropriate assessment, treatment and care, the current study was not designed to address these individual factors or outcomes. This study shows that an increase in tic symptoms is not ‘a whole group phenomenon’ and may be related to different or more sophisticated factors than we measured in this research.

## Summary

Previous research exploring the impact of COVID-19 on tics has been limited to anecdotal account and has not compared independent rated tic scores pre and during the pandemic. Using data from a randomised controlled trial (ORBIT-Trial), this study aimed to explore the impact of COVID-19 on tics. The study compared changes to scores on the YGTSS for two groups of participants, one group was followed up to 12 months’ post randomisation with no impact of COVID, and the other group was impacted by COVID-19 at the 12-month follow-up (during COVID group). The study found no differences in tic symptom or severity between those participants who were assessed before and during COVID-19. Further analysis revealed this was not influenced by age or gender, or baseline symptoms of anxiety or symptoms of ASD. Therefore, using pre and during pandemic scores we found no evidence that the COVID-19 pandemic had a significant impact on the symptoms of a group of young people with existing tic disorders. The sample was predominately male and thus more research in this area, particularly with females, is needed in order to better understand the impact and best support young people with tics.
